# Supplementary material for: Asymmetrical estimator for training encapsulated deep photonic neural networks
Source: Nat Commun. 2025 Mar 3;16:2143. doi: 10.1038/s41467-025-57459-5 (PMC11876688; doi:10.1038/s41467-025-57459-5)
Supplement: Supplementary file 1 — Supplementary Information [file 41467_2025_57459_MOESM1_ESM.pdf]

Supplementary information for "Asymmetrical estimator for training encapsulated deep photonic neural networks"

Yizhi Wang[1], Minjia Chen[1], Chunhui Yao[1, 2], Jie Ma[2], Ting Yan[2], Richard Penty[1], Qixiang Cheng[1,2*]

[1] Centre for Photonic Systems, Electrical Engineering Division, Department of Engineering, University of Cambridge, Cambridge, CB3 0FA, UK

[2] GlitterinTech Limited, Xuzhou, China

[*] Corresponding: qc223@cam.ac.uk

Supplementary Note 1. List of equipment used in the experiments:

For the experiments in this work, we chose wavelengths near 1550 nm in the C-band. Two types of laser sources have been used in the experiments. We used Thorlabs C-band tunable laser TLX1 and used ILX Lightwave 7900B system with 8 channels of 79800D precision fiber optic source modules. The readout PDs used in the experiments are HP 8153A with 81531A and 81536A modules. The EDFAs used in the experiments are the Connet MARS C-band pre-amp fiber amplifiers.

The modulation of input optical signal to the chip is applied via variable optical attenuators and optical modulators through arbitrary wave generators. The modulation units used in the experiments are Thorlabs V1550A and Thorlabs LNA6213. The two AWGs used in the experiments are Tektronix AFG3102C and Agilent 33500B.

The thermos-optic shifters with heaters are controlled by the voltage added. The voltage applied is controlled by an Arduino Mega 2560 with EVAL-AD5370 board. The output voltage range of 0-8V is amplified to 0-32V by a customized driver circuit board. The power supplies used are EL302D Dual Power Supply units. ILX Lightwave LDC-3724B controller is used for thermal control.

The optical signals on and off the chip are connected to the fibers via edge coupling to a 20-channel fiber array. Polarization controllers and tunable filters are used.

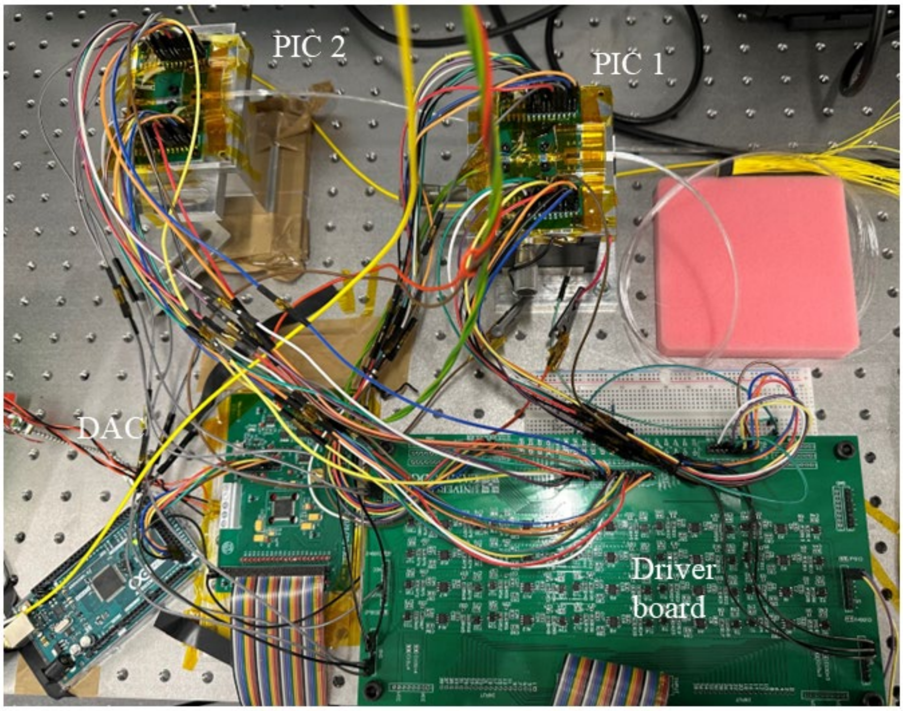

Supplementary Fig.1 The setup of the encapsulated DPNN demonstration. A picture showing the two PICs used for the experiment and the control system.

Supplementary Note 2. Experimental setup for subsection "AsyT method for training fully encapsulated PNNs"

For demonstrating AsyT's ability to train a fully encapsulated network, we utilize two copies of the SiN PIC to represent two layers of connections in a deep encapsulated network. The two chips are directly connected via a high-current density EDFA. The setup of the two chips can be found in Supplementary Fig.1. We can see the schematic of the system from Fig.2 in the main article. The signal input is from a tunable laser source with 10 dBm output centered at 1550 nm. The modulation to the input (sample) is applied through the variable optical attenuator. The attenuators have roughly -30 dB extinction ratio, which is sufficient for our experimental setting of discretization into 100 levels (see Methods of the main article).

The modulated signal representing the sample input is passed through a set of polarization controllers before entering the chip. The PIC is sensitive to polarization. Due to our signal power encoding of the neuron activation, we vary the PCs to find the position for the highest power. The controls are applied to the PIC through a DAC and driver circuit board with scripts programmed in Matlab. See the Results section of the main article for description of the estimation profile to apply the physical parameters to the system.

The signals into and out of the chip are edge coupled through the fiber arrays. After the propagation of signal through the first chip, the output signal is passed into the EDFA. We know that the gain of the EDFA can reach saturation and give a non-linear response when the set current density is large. Due to the propagation loss through the various components, we need to be aware of the insertion loss incurred after the first PIC. Consequently, the first set of EDFAs act as both the amplification and non-linear response of the system.

The second PIC system will also incur insertion loss on the propagation of the optical signal. As a result, we employ a second set of EDFAs with a lower current setting for a constant gain of linear response. The characterized difference in response with low and high current EDFAs can be found in Supplementary Fig.2. The arrangement of the two sets of EDFAs can be seen in Fig.2 (b) of the main article.

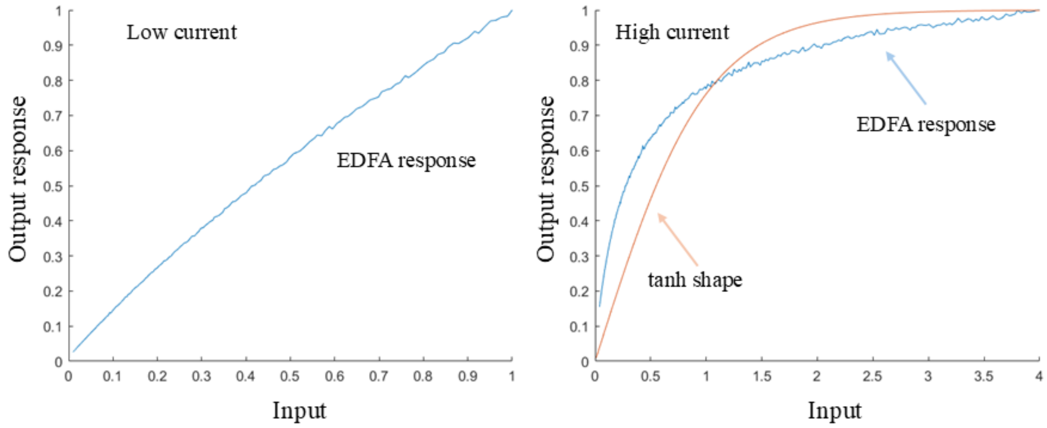

Supplementary Fig.2 The response with low and high current EDFAs. When a low current density is used, the response from the EDFA is relatively linear. When a high current is used, non-linear response similar to a tanh shape can be observed.

The output from the EDFA is usually a broadband signal. After the first sets of EDFAs (non-linearity), we use tunable filters centered around 1550 nm to filter out the undesired wavelengths. Before the signal is sent to the second PIC, polarization controllers are used again to control the polarization.

The second set of EDFAs are solely for amplification of a linear response. Due to the PDs' setting to center around 1550 nm, we don't need another set of tunable filters before detection. The neuron information at the output layer is then passed to the training system in the digital domain for computing the AsyT estimator and applied to the physical controls of the two PIC devices.

This setup encapsulates the optical signal without extracting it at the intermediate hidden neurons, serving as the demonstration that AsyT can train fully encapsulated systems. In comparison, the performance of the in-silico BP training and transfer sees significant performance degradation (see Fig.2 (e) of the main article).

Supplementary Note 3. Experimental setup for subsections "AsyT for training DPNN with only output neuron information" and "AsyT's compatibility with scale-up techniques"

The compatibility of AsyT to construct fully encapsulated deep PNN structures for fast training relies on AsyT's ability to train a deep PNN system with only the output neuron information. We further demonstrated two training tasks with the PIC device to experimentally validate this.

A similar setup for the sample input and PIC control are employed as the encapsulated experiment. Due to the equipment limitations, we instead add the non-linearity digitally to the DPNN system. To ensure that the non-linear response added is a sensible representation of photonically plausible non-linearity, we use the shape of the non-linearities in existing literature[1,2]. The sigmoid-like shape of the MRR response is illustrated in Supplementary Fig.3.

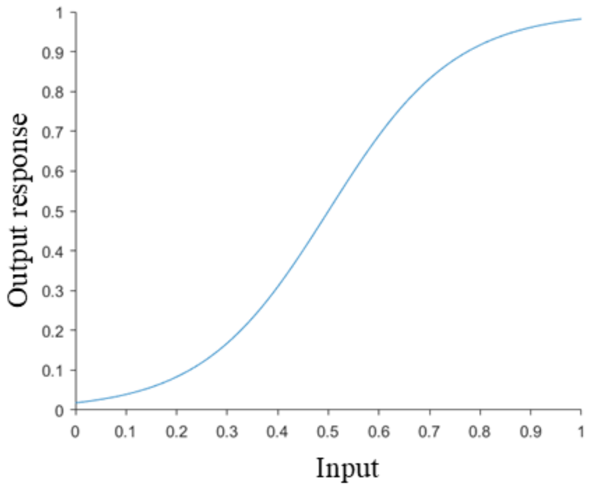

Supplementary Fig.3 The sigmoid-like response of the non-linearity added to the experiments for training with only the output neuron information.

To rigorously represent the non-idealities encountered in the construction of a fully encapsulated deep PNN, we deliberately add further error to the output response of the digital non-linearity. The error is added as a randomized normal distribution with the standard deviation of 0.1 for the output:

$$\frac{1}{\sigma\sqrt{2\pi}}e^{-\frac{1}{2}\left(\frac{x-\mu}{\sigma}\right)^2}$$

Despite the non-linearity is added digitally, the training system doesn't get access to the hidden layer neuron information at any stage of the training. The other parts of the experimental setup for these two subsections are the same as the demonstration for the fully encapsulated deep network.

Supplementary Note 4. Packaging and topology of the 4 × 4 SiN PIC

For a clearer viewing of the broadcast-select topology of the chip used in the experiments, we include a schematic in Supplementary Fig.4 (a). The enlarged photo of the chip can be found in Fig.2 (c) and (d) of the main article. A close-up photo of the packaging for the chip, including the wire-bonding, edge-coupling, and thermal controller is included in Supplementary Fig.4 (b).

As described in the main article, the information at four input channels from the previous layer is split into four copies by the two-stage 1×2 MMIs and accumulated by the two-stage 2×1 MMIs before entering the

representation of the next layer. There are 16 MZI unit cells on each PIC, which are all tuned by the same estimation profile.

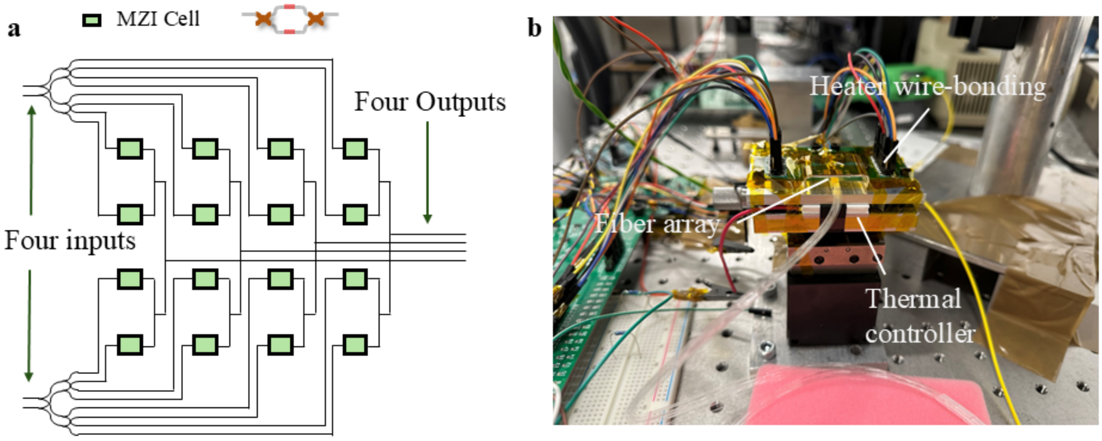

Supplementary Fig.4 The topology and packaging of PIC used in the experiments **a**, A schematic of the topology for the chip used in the experiment. **b**, A close-up photo of the packaging for each individual PIC.

Supplementary Note 5. Estimation profile through the theoretical analysis of the MZI

Each MZI cell is constructed by a stack of 1×2 MMI, a pair of thermos-optic phase shifters, and a 2×1 MMI. This stacked structure is also the basis for our estimation profile of the MZI unit. The ideal transfer matrix for the combined structure is:

$$\begin{pmatrix} \tau & j\kappa \\ j\kappa & \tau \end{pmatrix} \begin{pmatrix} e^{j\Delta\phi} & 0 \\ 0 & e^{-j\Delta\phi} \end{pmatrix} \begin{pmatrix} \tau & j\kappa \\ j\kappa & \tau \end{pmatrix}$$

Starting with an idealistic construction of the MZI unit, the splitting ratio is perfectly 50:50. The perfect splitting replaces τ and κ with ½. The expression can therefore be simplified to a sine and cosine expression as:

$$j \begin{pmatrix} \sin \Delta\phi & \cos \Delta\phi \\ \cos \Delta\phi & -\sin \Delta\phi \end{pmatrix}$$

The power transmission would indicate the expression as a cosine function:

$$T = \frac{P_{\mathrm{out}}}{P_{\mathrm{in}}} = \frac{1}{2}(1 + \cos 2\Delta\phi)$$

The expression is now for a given phase shift, but we would want to make some connection to actual parameters of the tunable physical system. The controlled parameter for our system is the voltage applied to the heaters for changing the effect path length ΔL. We know the phase shift is associated with the power applied to the system, given by $I \cdot V$. While the resistance might undergo some change with the heating, we can regard that as part of our knowledge distortion of mapping control. We thus reform the expression with only the parameters controllable by us as $\Delta\phi \propto V^2$.

$$T = \frac{1}{2}(1 + \cos(2\gamma^{\star} \mathrm{V}'^2 + \mathrm{C})) + T_{\mathrm{base}}$$

A detailed description of the realistic system would include the consideration of a few more terms, such as C determining the initial state and the extinction ratio with T_{base}. However, these are not essential for our estimation profile, because we only need a very basic description of the system which leads to simplified form seen in the main article as:

$$T = \frac{1}{2}(1 + \cos(2\gamma^{\star} \mathrm{V}'^2))$$

Supplementary Note 6. Additional estimation profiles used for obtaining the results in "AsyT for training DPNN with only output neuron information" and Fig.3 (d)

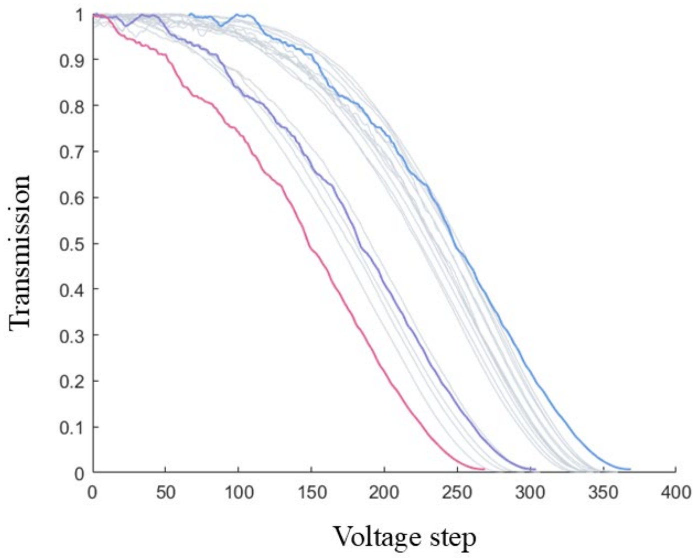

Supplementary Fig.5 The three additional estimation profiles used for the obtaining the results in Fig.3 (d) of the main article.

For validating the generality of the estimation profile used for the experiment, we repeated the training with three additional estimation profiles. Supplementary Fig.5 shows three additional estimation profiles which are used to obtain the results in Fig.3 (d). The estimation profile 2 (purple line in the center of Supplementary Fig.5) is based on a single sampling of the transmission behavior in the chip 2 when not used for the experiment of subsections "AsyT for training deep PNN with only output neuron information" and "AsyT's compatibility with scale-up techniques".

The other two profiles are obtained through shifting the transmission behavior of profiles to extreme levels beyond the boundaries of the characterized transmission shapes (the two lines on the left and right of Supplementary Fig.5). As shown in Fig.3 (d) of the main article, the training performance is maintained for all the estimation profiles used, demonstrating that different estimation profiles can be used to achieve the same high-level performance with AsyT. AsyT can allow training as long as the estimation profile has any statistical relevance to the actual control profile.

Supplementary Note 7. More discussion on the concept of AsyT and why pure BP or DFA wouldn't be sufficient for training

The sensitiveness of BP to error is commonly known[3], the deviation between the expected transformation $f_m(\cdot)$ and the resulting physical transformation $f_p(\cdot)$ degrades the training performance. The insufficiency of in-silico BP and transfer has been discussed in the main article, where the results in Fig.2, 3, 4, 5 all show significant degradation of the in-silico BP accuracy. We have also shown that the output neuron information alone can't suffice training for existing BP-based methods which only have the digital model in the backwards pass in Fig.3 (**a**).

One part of the training with AsyT is the alignment of the physical system's transformation towards the digital parallel model. However, the simple concept of alignment by itself isn't sufficient for training PNNs, instead it must be combined with BP update in the digital model. The criterion of alignment-based methods[4,5] such as DFA requires an additional set of random fixed matrices which the error matrix **E** is multiplied by and directly fed to the layers as the updates. The original criterion of DFA relies on the fact that the multiplication with the fixed matrix is statistically aligned with the actual gradient update to the system. However, this criterion is only true for

digital systems where the resulting transformation $f_m(\cdot)$ is clearly describable with the parameter W_{set} set to the system. For physical systems in PNNs, the resulting transformation of $f_g(\cdot)$ can't be accurately described by the parameter input W_{set}. We have shown in the Methods section that the transformation in two domains at every time instance can be described as a projection $f_p^t(\cdot) = \mathbf{D}^t f_m^t(\cdot)$. For the set of random fixed matrices in DFA defined as $\mathbf{B}$, we see that the directly updating the system with the error matrix as DFA leads to a multiplication in the direction of $\mathbf{D}^t\mathbf{B}$ for each iteration. Multiplication to $\mathbf{E}$ is no longer based on a set of fixed matrices, thus leading to breakage of the alignment criterion for DFA. A simulation showing the performance degradation of pure DFA training on the PNN is shown in Supplementary Fig.6.

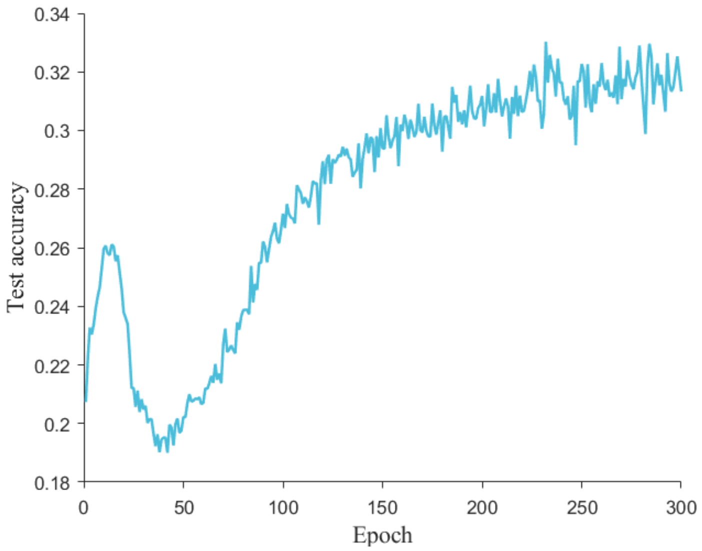

Supplementary Fig.6 DFA by itself is not sufficient for training erroneous DPNNs, resulting in significant performance degradation.

While AsyT leverages some concepts of alignment, AsyT is more associated with the BP update dynamics and the concept of gradient updates. AsyT is a BP-based method in which the alignment of the transformation serves as the proxy of the actual BP gradient update to the physical control parameters at each time instance. The alignment process can be viewed as a self-modulated mechanism for correcting the mismatch of transformations to allow AsyT's convergence towards ideal BP. This shows the necessity of employing the additional forward pass in the digital parallel model.

$$\Delta\mathbf{W}_{\mathrm{AsyT}}^{[l]} = M_{w1} \cdot \Delta\mathbf{W}_{\mathrm{dig}}^{[l]} + M_{w2} \cdot \Delta\mathbf{W}_{\mathrm{pseudo}}^{[l]}$$

As shown by the expression of the AsyT estimator, when the mismatch between the expected and actual transformations is small towards the end of the training, the expression of the AsyT estimator converges towards the ideal BP expression with the requirement of $M_{w1} + M_{w2} = 1$.

Supplementary Note 8. Analysis on the mixing ratio of M_{w1} and M_{w2}

For most scenarios, it is sufficient to set the two mixing ratios as equal contributing of $M_{w1} = M_{w2} = 0.5$. This setting allows for equal contribution of the information from the physical system and digital parallel model. From the simulation study, we see that the training can be sufficed with different mixing as long as the mix is not one of the extremes where only one contribution remains (i.e. $M_{w1} = 1$ or $M_{w2} = 1$). The result for varying M_{w1} is illustrated in Supplementary Fig.7. All mixings apart from $M_{w1} = 0$ and $M_{w1} = 1$ can allow for training, with the equal contributing of $M_{w1} = M_{w2} = 0.5$ resulting in the highest performance.

As described in the subsection of "Asymmetrical training method", the procedure for implementing AsyT includes maintaining the learning rate of the digital update and the overall AsyT estimator the same. The learning rate α itself can vary for different update instance t, but must be maintained the same for update to the two sets of parameters at a given time. The fixed mixing ratio during the training acts as a self-regulating process for compensating for the effect of magnitude difference from the digital domain and the physical system. When one

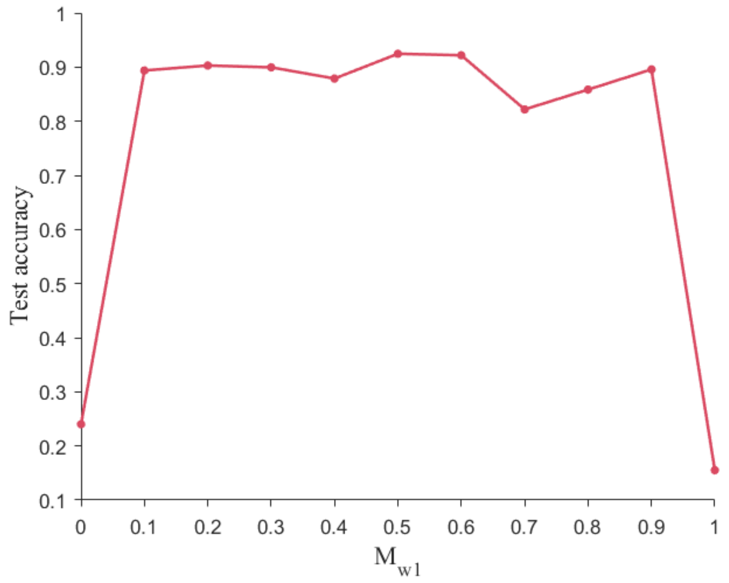

Supplementary Fig.7 Performance obtained when changing the mixing ratio in AsyT, $M_{w1} = 1$ corresponds to the pure in-silico BP training and $M_{w1} = 0$ corresponds to training only with the pseudo update.

of the errors is larger than the other, we see that the overall magnitude of the AsyT estimator update $\alpha\Delta\mathbf{W}_{\mathrm{AsyT}}^{[l]}$ will be different from the digital parallel model update of $\alpha\Delta\mathbf{W}_{\mathrm{dig}}^{[l]}$. This means that when the physical system is further away from the loss function minimum than the digital model, the update tries to catch up with the training progress of the digital model. On the other hand, when the physical transformation is at a position which is closer to the minimum, the AsyT estimator slows down the process of update for retaining the relevance between the digital and AsyT updates.

The physical boundaries of the PNNs again help with preserving the robustness of AsyT (see the Methods section in the main article for first part of the discussion). If there is a significant magnitude difference between the two contributing parts of $\Delta\mathbf{W}_{\mathrm{dig}}^{[l]}$ and $\Delta\mathbf{W}_{\mathrm{pseudo}}^{[l]}$, the overall AsyT estimator might lose relevance to the digital parallel model. However, for the bounded system of PNNs, the allowed magnitude deviation is restricted by the physical boundaries as discussed in the Methods section. The training is thus well-behaved for any statistical deviation of the physical module.

Supplementary Note 9. Simulation analysis of AsyT

The relevant datasets used in the simulation can be found in the Sci-kit learn[6] and PyTorch[7] libraries. The simulation serves as a general-purpose representation of scalable isomorphic PNNs on different platforms. Consequently, rather than thinking about the physical errors limited to the photonic platforms, we implement the physical pass with a more generic description. The expression of the simulated physical transformation is given as follows.

$$f_p^t(\mathbf{W}^t) = \left(\mathbf{I} + \mathbf{P}_{\mathrm{sys}}^t\right) \otimes \left(f_m^t(\mathbf{W}^t) + \mathbf{N}_{\mathrm{init}}\right) + \mathbf{N}_{\mathrm{rand}}^t$$

We can equally think of the physical transformation as a deviation from the mathematical transformation of the digital model as the equation above. For determining the experiment-level error to be added to the simulation, we use the characterized transmission behaviors of the MZI units. As illustrated in Fig.2 (f) of the main article, the highest deviation in the transmission representation occurs at the normalized voltage of 0.5, where the maximum transmission value represented is roughly 0.9, and the minimum represented is roughly 0.3. This means that we can form an overestimation of the deviation from the center of these two extreme cases as a roughly $\pm 60\%$ deviation in terms of transmission value encoding. This general deviation is then treated as 1 σ_{phy}.

For an overestimation of the deviation, we assume that this deviation to be true for the entire tunable range, except for the cases where the physical boundaries limit the further deviation. We utilize a normal distribution for constructing this systematic deviation as a mask of $\mathbf{P}_{\text{sys}}^{t}$. As we know that the physical systems always have a baseline encoding value determined by the extinction ratio of the components, this is represented by the term $\mathbf{N}_{\text{init}}$ as the initial deviation to the boundary states represented by the individual components. This value is inherent to the specific physical component and is thus not varies as t. The last term we add is the random fluctuation term $\mathbf{N}_{\text{rand}}^{t}$ for representing the degradation of the signal due to the uncontrollable noises.

For simulating the case of realistic training with a sharp perturbation (Fig.5 (g)), we set the system to a new deviation state with $1.2 \times$ the deviation before the 20th epoch. For the very noisy low fidelity system, we add the randomized error $\mathbf{N}_{\text{rand}}$ with a standard deviation of 50% the value encoded, translating to an extreme case of mean SNR of 4. AsyT is still able to train under these extreme conditions, showcasing the added robustness and tolerance with the extra forward digital pass.

Supplementary Note 10. Discussion on the interface speed, propagation speed and propagation time of the AsyT encapsulated PNN

As described in the main article, we have defined the extraction time T_{extract} to be the minimum time required before sufficient information is available from the PNN structure for useful operation. More specifically, T_{extract} is the summation of the propagation time through the PNN device and the interface time required to readout that piece of information. One of the assumptions we have made in the discussion is that the propagation time is a much shorter time than the access/interface time. Here we discuss further the details of this argument.

For representation of a A node to A node connection, the most general hardware representation would be a fully connected $A \times A$ connection. If we define the hardware depth as the number of repetitive components that the signal must encounter before fully modulated for the $A \times A$ connectivity. We can realize that the minimum scaling to be A as a direct mapping topology. Even with other decomposed topologies[8–10], the scaling of the photonic hardware path grows linearly with the connectivity size. The propagation time of the in-transformation computation is directly proportional to the hard depth. If we take the SiN platform used in the experiments as an example, the refractive index is around 1.93[11]. The total on-chip waveguide length of the device is roughly 12.5 mm, corresponding to an on-chip propagation time of 0.08 ns. We can use this as an estimation of the basic propagation time for the hardware component as 0.02 ns per photonic depth.

In comparison, even when considering specialized serial communication protocol of JESD204B[12], with a data transmission rate of 12.5 Gbps. (Here we are considering the data transmission to be the bottleneck of the interface speed. Depending on the exact setup, it is also possible that the ADC sampling rate is the bottleneck.) For the control and readouts of 16-bits, we can expect a roughly 1.28 ns time cost for each access point. The scaling of the access point is described as $O(2M - P)$. Consequently, even when we ignore the internal data shuttling time the scaling of the interface time with respect to the hidden neuron number is over 100× (for the factor of 2) compared to the scaling of the propagation time. Thus, in the main article where relevant, the description of the extraction time scaling for the encapsulated deep PNN is defined as $O(P)$ for simplification. Furthermore, for the other more accessible protocol such as serial SPI communication, the data transmission rate is typically 60-100 Mbps (27 μs), creating an even larger gap between the propagation time and the interface time. We can see from the above analysis that the construction of an encapsulated DPNN can be time efficient compared to truncated ones.

Supplementary Note 11. Energy discussion for systems with distributed computational overhead

As formulated in the Discussion section, for multiple copies of the PNN devices, the computational overhead can be distributed by generally applying the same relevant digital model parameters to a series of PNN systems. For all the BP-based methods, the backwards pass computation for the gradient estimate is necessary. Methods such

as PAT and HT all require this computation step. In comparison, the additional digital computation of AsyT would be the forward pass in the digital model. Here we discuss how the computational overhead of the parallel digital model in AsyT can be distributed with respect to each local PNN copy for an overall reduced resource requirement that is similar to the standard BP process. The digital computation of a matrix multiplication operation with dimensions of $(n \times p)$ and $(p \times m)$ can be described as $nm(2p-1)$[13] FLOPs. The number of FLOPs for the backward pass to the forward pass takes a standard ratio of 2:1[14,15], where this ratio can be further reduced with the optimization of batch size. Suppose that the energy needed for completing the digital training is $E_{\text{Dig-BP}}$.

In AsyT, the parallel digital model for a certain network structure and task isn't limited to a specific copy of the PNN device. Instead, it would be a generally applicable model for PNN copies with variations. (Essentially, this means that digital updates acquisition is a standalone process that can be performed prior to the local training of a specific PNN copy.) This means that the more PNN copies are employed, the closer the distributed overhead is compared to standard BP. AsyT's property is in line with reproducible PNNs' goal for computation acceleration despite the device variation. For example, if there is a total of N PNN copies being manufactured, the actual computational overhead associated with each of the copy is only $E_{\text{Dig-BP}}/N$. Also, since all the PNN copies here in discussion are constructed with encapsulation, the individual operational and control overhead is reduced, allowing for an overall efficient operation.

Supplementary Note 12. Different types of PhyNN training methods and their applicational scenarios

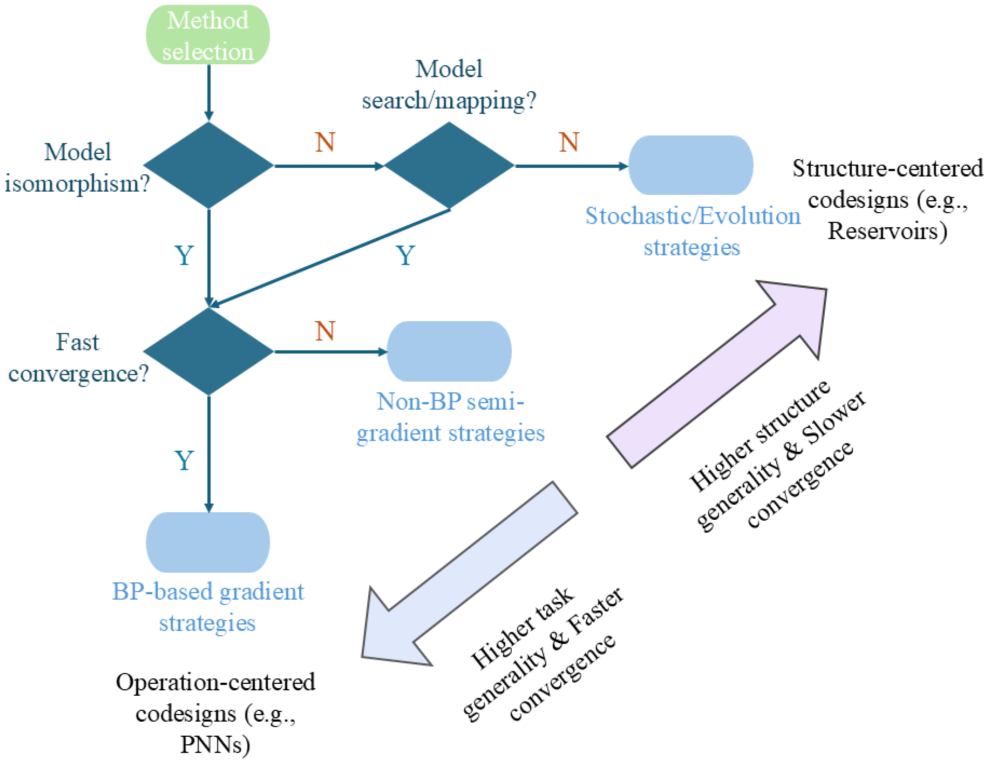

Supplementary Fig.8 A decision flowchart for choosing the appropriate training method category depending on the applicational scenario.

There are many different categories of PhyNN training methods. Each category of training methods has their own unique properties and advantages. Unlike mathematical (digital) NNs, constructing a PhyNN is often a codesign process between the physical device structure, the task, the applicational scenario, and the training algorithm used. Consequently, it is not sensible to regard a single class of training methods as the elixir for every type of PhyNN construction. Instead, it is more appropriate to understand that each category has its own appropriate application

scenarios. Here we present a summarized discussion on how different categories of training methods can be more suitable for different types of PhyNN codesign (implementation strategy)[16].

Supplementary Fig.8 shows a decision flowchart for choosing suitable methods for a specific PhyNN implementation technique. The construction/codesign of PhyNNs can be broadly divided into structure-centered ones and operation-centered ones. Structure-centered PhyNNs are typically the ones where the codesign logic is based on the attempt to map a device with high complexity to certain specific tasks. One great example of the structure-centered PhyNN is reservoir computing, where a device with high complexity (randomized spatial and temporal connectivity) is used for the ability to reduce a nonlinear training task to a linear separation problem. The structure-centered codesign often lacks isomorphism to a specific model description. Consequently, the common approaches are often stochastic or non-model based, such as particle swarm optimization and genetic algorithms.

On the other hand, operation-centered PhyNN implementations are cases where the codesign starts with the acceleration or partial network isomorphism in mind. PNNs are great examples of operation-based PhyNNs. For instance, the high bandwidth of photonic processing (through temporal and wavelength domain multiplexing) is often leveraged to construct PNNs which have high throughput. PNN's acceleration can most predominantly be found as the connectivity matrix (MVM operation) acceleration[17]. In these types of operation-centered, since the PhyNN device structure are designed with the goal of acceleration in mind, these systems often exhibit sufficient levels of isomorphism for a model/connectivity description to be projected onto the system. For example, in PIC PNN, information such as connectivity (transmission) is fundamentally determined by physical dimensions such as system topology, path length, heater resistance, fabrication tolerance, etc. Thus, it is more sensible to utilize this existing information well rather than deliberately discarding it for the equal treatment to structure-centered codesigns. For these cases, the most desirable category is often the gradient-based methods like BP for the overall fast convergence and generality.

Both codesign logics have their advantages and limitations. For example, the operation-centered codesigns come at a higher repeatability, task generality, and faster convergence (through the compatible training methods). These implementations are generally targeted at the popularization of PhyNN acceleration through reproducibility. PIC-based PNN is a great example where motivation lies in the repeatable acceleration for a wide range of users. On the other hand, structure-centered codesigns have a lower requirement of isomorphism. Yet, the trade-off would be the lower task generality, longer training convergence, and higher difficulty in repeating the system. Supplementary Fig.8 provides a concise decision flowchart for choosing the appropriate training method.

Supplementary Note 13. The alignment-inspired training methods: AsyT, PAT, and DFA

While methods such as AsyT, PAT[18], and DFA[4] are all technically alignment-inspired methods, the concept of alignment is slightly different for each of these methods. Here we describe the difference between these alignment-inspired methods and thus discuss the convergence logic of AsyT.

To start with, AsyT and PAT would be categorized into BP-based methods while DFA is alignment-based method. The difference in the categorization originates from the update dynamics of these methods. For example, both AsyT and PAT utilize the BP-like layer-by-layer update logic, in which the error at the output layer is backwards fed to optimize for the loss with respect to each layer. On the other hand, DFA's update to each layer is obtained through the multiplication between the error matrix and a set of random fixed matrices. Thus, error optimization is a process with respect to the entire deep network structure. (See Supplementary Fig.9 for the comparison between the update dynamics.) This is also the reason why increasing the network complexity through adding deep layers often result in a less increased performance for DFA compared to BP. The layer-by-layer gradient optimization is the fastest loss reducing operation, which grants BP the faster convergence compared to DFA.

Consequently, despite all being alignment-inspired methods, the BP-based ones and non-BP-based ones still exhibit significant differences. For the two BP-based methods of AsyT and PAT, we also need to be aware of how each method leverages the concept of alignment differently. For PAT, digital backwards pass grants a gradient

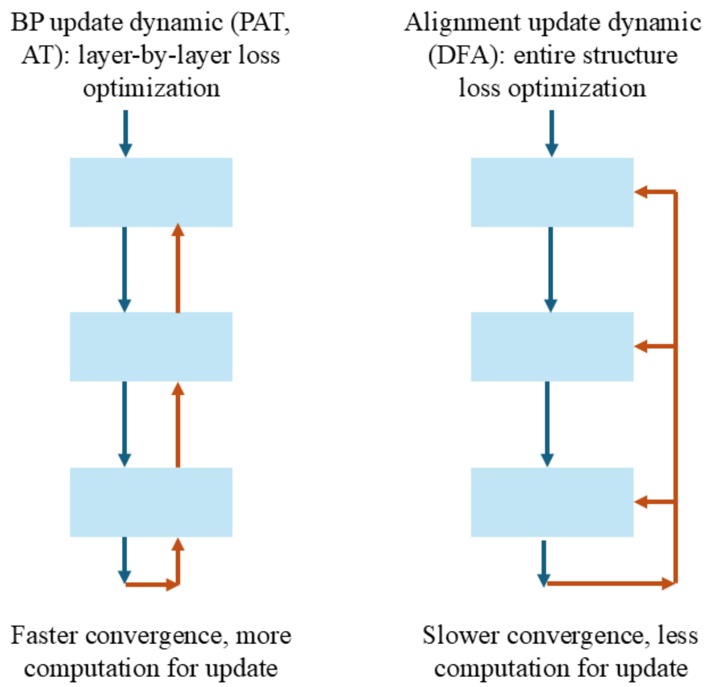

Supplementary Fig.9 The difference in update dynamics between the BP-based methods (PAT, AsyT) and alignment-based methods (DFA).

estimator to the control updates. However, since the control-transformation mapping isn't perfect information for PAT, the update estimator is essentially a relaxed condition of alignment between the actual physical gradient $\delta \mathbf{W}_{\mathrm{phy}}$ and the PAT update $\Delta \mathbf{W}_{\mathrm{PAT}}$. If these two directions are within 90°, then we know the update behavior must be loss-reducing. On the other hand, AsyT's parallel digital model is a complete standalone forward-backward process trained directly by digital BP. This means that we know the loss behavior for the digital model in AsyT must be converging. Therefore, the utilization of the alignment concept is between the resulting digital update direction $\delta \mathbf{W}_{\mathrm{dig}}$ and the AsyT update direction $\Delta \mathbf{W}_{\mathrm{AsyT}}$. We know that $\delta \mathbf{W}_{\mathrm{dig}}$ is a loss-reducing direction; so, within the alignment of 90°, the AsyT update $\Delta \mathbf{W}_{\mathrm{AsyT}}$ must also be a loss-reducing direction. This is the reason why we stated that while the learning rate can be time dependent as $\alpha(T)$, it must be the same for the digital and photonic model update within one epoch (see Results section). We can recognize that the overall update is regulated by the digital update (a direction that we know is converging), thus controlling AsyT's convergence rate with the BP behavior. While both $\delta \mathbf{W}_{\mathrm{phy}}$ and $\delta \mathbf{W}_{\mathrm{dig}}$ are directions with loss-reducing behavior, we can note that $\delta \mathbf{W}_{\mathrm{phy}}$ is purely a local direction specific to the PNN copy while $\delta \mathbf{W}_{\mathrm{dig}}$ is general direction that is applicable to multiple PNN copies. Consequently, it leads to the analysis in the main article's Discussion section on the generality of a single digital model in AsyT for multiple PNN copies to achieve the reduced distributed computational overhead.

Supplementary Note 14. The trade-off between gradient-based (BP) and non-gradient-based (FS) methods

Finite stochastic (FS) methods[19] are non-gradient-based methods often used for training. While BP offers faster convergence than FS, FS methods do have a lower update computation complexity compared to BP. However, in the implementation of PhyNNs, the overall efficiency should consider the overall consumption of the system rather than only the computation of the update. Since the exact efficiency of each method would be highly dependent on the task (due to the varying complexity and convergence behavior of the task), it would be impossible to construct an exhaustive list discussing all the existing machine learning tasks. Instead, here we formulate a generic discussion using BP and FS methods as example to highlight some of the aspects that the users should consider when deciding which category of training method would be the best for them.

We know that all PhyNNs are hybrid digital-analogue systems. Even the simplest ones that don't require digital models for training still need digital system for the parameter controls. For each iteration, we can formulate the total energy requirement as follows for BP and FS PNNs.

$$E_{\text{BP-PNN}} = P_{\text{photonic}} \cdot t_{\text{opt-BP}} + E_{\text{BP}} \cdot I_{\text{BP}} + E_{\text{interface}} \cdot I_{\text{BP}}$$

$$E_{\text{FS-PNN}} = P_{\text{photonic}} \cdot t_{\text{opt-FS}} + E_{\text{FS}} \cdot I_{\text{FS}} + E_{\text{interface}} \cdot I_{\text{FS}}$$

P_{photonic} represents the operating power of the photonic device (that is, including the component control, input signal power, etc.). $t_{\text{opt-XX}}$ is the total operational time required for each method. $E_{\text{BP/FS}}$ is the energy required to compute the update for each iteration. $I_{\text{BP/FS}}$ is the number of iterations required for a specific convergence level. $E_{\text{interface}}$ is the energy required at the interface for setting the new parameters and readouts between each iteration.

Since the photonic structure of FS and BP (via AsyT) can both be constructed as encapsulated networks, we can assume that the power consumption of the two systems to be roughly the same. We know that due to the simplicity of the FS update computation, the energy required for computing each update would be lower than the BP computation as $E_{\text{FS}} < E_{\text{BP}}$. Nonetheless, the natural trade-off comes with the easier computation is the slower convergence and longer operation time, $I_{\text{BP}} < I_{\text{FS}}$ and $t_{\text{opt-BP}} < t_{\text{opt-FS}}$. Depending on whether the photonic or the digital system is the more power-hungry side, one method might be more efficient than the other for a specific case. For example, in complex tasks (large I_{XX} and high $t_{\text{opt-XX}}$ baseline) with a large network structure (high power consumption of P_{photonic} and large number of A-D control interfaces, i.e. high $E_{\text{interface}}$), fast convergence is a top priority so BP methods might be more favorable. It is very likely that the exact more efficient method would vary for different combinations of PNN platform, equipment, and the digital system's specifications.

Therefore, the above analysis again shows that no single category of training method is the elixir for all types of PhyNN implementation. Depending on the end goal and the applicational scenario (also the available equipment), it is advised that the user should choose the appropriate method by considering the overall PhyNN construction rather than assuming a single category as the best.

Supplementary Note 15. Discussion on AsyT for diffractive PNNs

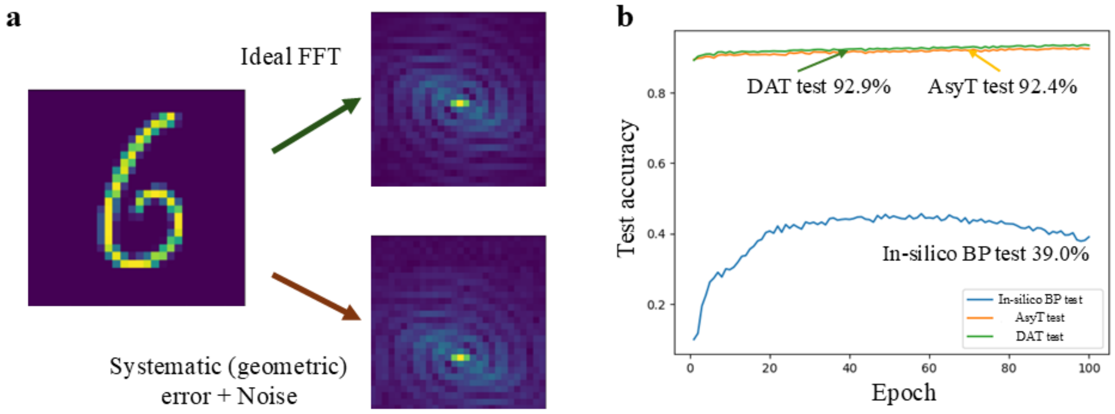

Supplementary Fig.10 The simulation of AsyT for spatial PNNs. **A**, In realistic implementations, the system might suffer from geometric and fabrication errors. **b**, AsyT shows significant improvement in performance compared to in-silico BP. AsyT can achieve comparable performance with lower computation complexity.

AsyT trains the non-ideal physical structure by interpreting its general mathematical transformation description towards the parallel model, which means the generality of application on different PhyNN platforms with reasonably describable isomorphism. Here we discuss the compatibility of AsyT with diffractive PNNs through simulation to showcase the generality of AsyT method's application. The construction of the diffractive PNN includes an input controlled by a digital micro-mirror device (DMDs) for tuning each pixel of the input image. The input signal is then passed through a lens which acts as a two-dimensional FFT on the image. The Fourier

transformed signal is modulated by spatial light modulators (SLMs). The combination of the modulating blocks is viewed as the deep network structure of the PNN. On top of the systematic knowledge imperfections (such as due to the non-ideal fabrication and control system), we add the effect of geometric error to the system (as the form of signal shift). For example, when a translational shifting acts on the PNN system, the resulting system experiences both a systematic error and some loss in the information (see Supplementary Fig.10 (a), where part of the signal is assumed to be outside of the detection area of the CCD camera). Similar to the case of the integrated PNNs, at each place of tuning (i.e. applying the physical control parameters), the mismatch between how the user interprets the transformation and the actual physical transformation is fundamentally resulting from imperfect fabrication and system construction. Random noise is also added to the input image to emulate the undesired light sources in realistic implementations. For investigating the ability of AsyT to train imperfect spatial PNN, we utilize the geometric error of five pixels' shift. The results are shown in Supplementary Fig.10: in-silico BP training suffers a significant degradation in performance, with the test accuracy for the MNIST task is reduced to 39%. On the other hand, when AsyT is used for PNN training, test accuracy is improved to 92.4%, showing significant performance improvement. AsyT's generality theoretically allows the training to be conducted to different PhyNN implementation platforms.

Supplementary Note 16. AsyT as a lightweight and efficient option for training reproducible DPNNs

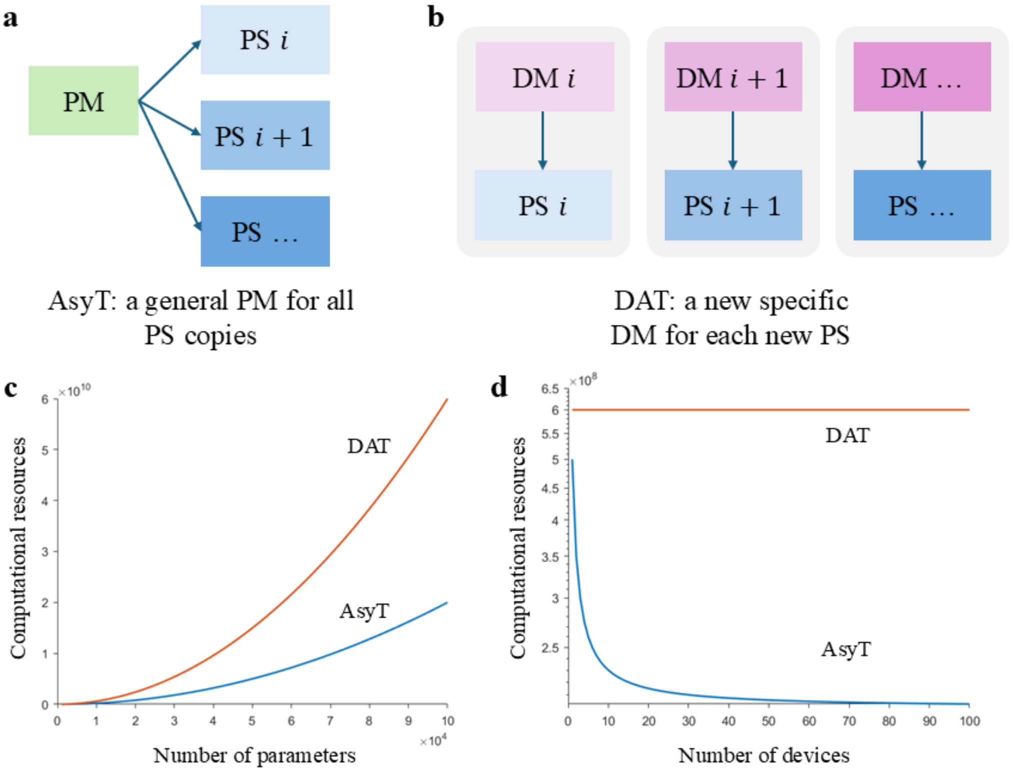

Supplementary Fig.11 AsyT is optimized for reproducible DPNNs. **a**, **b**, For reproducible copies of the PNN system, AsyT applies a single PM generally to all the PS copies, whereas alternative methods have device specific digital model construction which can add computational complexity and reduce efficiency. **c**, **d**, AsyT requires lower computational resources. PM: Parallel Model; PS: Photonic System; DM: Digital Model.

AsyT is designed to be a lightweight PNN training solution with the efficiency of training reproducible PNNs, which is essential for the popularization and commercialization of PNN accelerators. As mentioned in S12, different training methods each have different application scope. Compared to methods whose application scope is leaning towards the benchtop setting in a designated environment (and where the users are typically not concerned with the resources associated with the training process), AsyT has lowered computational complexity

and resource requirement. The computation efficiency of AsyT originates from both the training of each device due to the simplicity of the parallel model, and the training of multiple reproducible PNN copies due to the generality of the parallel model's updates. Taking the DAT[20] as an example for comparison, the digital model in DAT is a detailed simulation of the photonic system, in which the information transformation is encoded by the specific photonic system's characteristics ($S_n = |S_n|\, exp(j\Phi_{S_n})$). In comparison, AsyT's parallel model is considered as a simple mathematical transformation, as is in the case of a digital forward propagation. This allows AsyT to save computational resources for the creation of the digital side. Furthermore, the similarity loss and task loss both need to be optimized with respect to the physical parameter in DAT. In comparison, only the task loss needs to be backpropagated for AsyT, allowing for significantly less resource associated (Supplementary Fig.11 (c)) when number of parameters in the network grows. Furthermore, since a global parallel model in AsyT can be applied to different copies of local PNN devices, the computation overhead can be distributed for multiple devices (Supplementary Fig.11(d)). The overhead associated with each local PNN copy is significantly reduced while the resource requirement for each copy in DAT can't be distributed. (Here the computational resources required is estimated by considering the number of FLOPs where the number of operations is scaling with number of parameters. The number of operation in the backward pass is estimated to be twice the forward pass[15].) For the same error setting, AsyT can achieve comparable performance to alternative methods (92.4% vs 92.9%, Supplementary Fig.10 (b)) while significantly reducing the computational resources required (and thus the computation time for the updates). These factors show the advantages of utilizing AsyT for training reproducible PNN devices (such as based on integrated photonics). The lowered computation requirement for the complementary system also shows potentials for integrated device close to the native data generation source, paving way for upcoming computation schemes such as edge computing with photonic networking.

Reference:

1. Rausell Campo, J. R. & Perez-Lopez, D. Reconfigurable Activation Functions in Integrated Optical Neural Networks. *IEEE J. Select. Topics Quantum Electron.* **28**, 1–13 (2022).
2. Xu, Z. *et al.* Reconfigurable nonlinear photonic activation function for photonic neural network based on non-volatile opto-resistive RAM switch. *Light Sci Appl* **11**, 288 (2022).
3. Lillicrap, T. P., Santoro, A., Marris, L., Akerman, C. J. & Hinton, G. Backpropagation and the brain. *Nat Rev Neurosci* **21**, 335–346 (2020).
4. Nøkland, A. Direct Feedback Alignment Provides Learning in Deep Neural Networks. Preprint at http://arxiv.org/abs/1609.01596 (2016).
5. Lillicrap, T. P., Cownden, D., Tweed, D. B. & Akerman, C. J. Random synaptic feedback weights support error backpropagation for deep learning. *Nat Commun* **7**, 13276 (2016).
6. Pedregosa, F. *et al.* Scikit-learn: Machine Learning in Python. *MACHINE LEARNING IN PYTHON* (2011).
7. Paszke, A. *et al.* PyTorch: An Imperative Style, High-Performance Deep Learning Library. Preprint at http://arxiv.org/abs/1912.01703 (2019).
8. Flamini, F. *et al.* Benchmarking integrated linear-optical architectures for quantum information processing. *Sci Rep* **7**, 15133 (2017).

9. Clements, W. R., Humphreys, P. C., Metcalf, B. J., Kolthammer, W. S. & Walsmley, I. A. Optimal design for universal multiport interferometers. *Optica* **3**, 1460 (2016).
10. Reck, M., Zeilinger, A., Bernstein, H. J. & Bertani, P. Experimental realization of any discrete unitary operator. *Phys. Rev. Lett.* **73**, 58–61 (1994).
11. Xiang, C., Jin, W. & Bowers, J. E. Silicon nitride passive and active photonic integrated circuits: trends and prospects. *Photon. Res.* **10**, A82 (2022).
12. Texas Instrument. JESD204B Overview. (2016).
13. López, F., Karlsson, L. & Bientinesi, P. FLOPs as a Discriminant for Dense Linear Algebra Algorithms. in *Proceedings of the 51st International Conference on Parallel Processing* 1–10 (2022). doi:10.1145/3545008.3545072.
14. Paria, B. *et al.* Minimizing FLOPs to Learn Efficient Sparse Representations. Preprint at http://arxiv.org/abs/2004.05665 (2020).
15. Wang, Z., Chen, J. & Zhu, J. Efficient Backpropagation with Variance-Controlled Adaptive Sampling. Preprint at http://arxiv.org/abs/2402.17227 (2024).
16. Momeni, A. *et al.* Training of Physical Neural Networks. Preprint at http://arxiv.org/abs/2406.03372 (2024).
17. Zhou, H. *et al.* Photonic matrix multiplication lights up photonic accelerator and beyond. *Light Sci Appl* **11**, 30 (2022).
18. Wright, L. G. *et al.* Deep physical neural networks trained with backpropagation. *Nature* **601**, 549–555 (2022).
19. Bandyopadhyay, S. *et al.* Single chip photonic deep neural network with accelerated training. Preprint at http://arxiv.org/abs/2208.01623 (2022).
20. Zheng, Z. *et al.* Dual adaptive training of photonic neural networks. *Nat Mach Intell* (2023) doi:10.1038/s42256-023-00723-4.
